# Outcomes of Cardiac Arrest and Resuscitation in the Emergency Department: A Retrospective Study at a Tertiary Care Center in South India

**DOI:** 10.7759/cureus.88646

**Published:** 2025-07-24

**Authors:** Rajarajeswaran Krishnan, Hari Prasad, Dhileeban CM, Deebak Raaj, Prahadheeshwar K, Ashni Bhandari

**Affiliations:** 1 Emergency Medicine, SRM Medical College Hospital and Research Centre, Chennai, IND; 2 Biostatistics and Epidemiology, Sree Balaji Medical College & Hospital, Chennai, IND

**Keywords:** cardiac arrest, cardiopulmonary resuscitation, emergency department, glasgow coma scale, in-hospital cardiac arrest, neurological outcome, post-arrest care, resuscitation outcome, return of spontaneous circulation, survival rate

## Abstract

Introduction: Cardiac arrest is a critical medical emergency associated with high mortality and variable neurological outcomes. Despite advancements in resuscitation practices, survival following cardiac arrest remains suboptimal in many healthcare settings. This study aimed to evaluate the clinical and neurological outcomes of patients who experienced cardiac arrest in the emergency department of a tertiary care center and to identify key factors influencing survival.

Methods: This retrospective observational study was conducted at SRM Medical College Hospital and Research Centre, Kattankulathur, between August 2024 and May 2025. A total of 240 patients with documented cardiac arrest that occurred in the emergency department were included. Of these, some were later transferred to the intensive care unit (ICU) for continued management. Data on demographics, arrest characteristics, initial rhythm, time to cardiopulmonary resuscitation (CPR), return of spontaneous circulation (ROSC), survival beyond 48 hours, neurological outcomes, and need for ventilatory support were collected and analyzed.

Results: Among the 240 patients included, 160 (66.6%) were male. Most cardiac arrests were witnessed events (n = 225, 93.8%), and all occurred in the emergency department (n = 240, 100%). A total of 107 patients (44.6%) were subsequently transferred to the ICU for ongoing care following initial resuscitation. The initial cardiac rhythm was non-shockable in 200 patients (83.3%) and shockable in 40 patients (16.7%). CPR was initiated within one minute in 156 patients (65.0%) and within two to three minutes in 84 patients (35.0%). ROSC was achieved in 154 patients (64.1%), and survival beyond 48 hours was observed in 154 patients (64.1%). Among the survivors, 111 (72.1%) had a Glasgow Coma Scale (GCS) score of 15/15 at discharge, while 17 (10.8%) required mechanical ventilation at the time of discharge.

Conclusion: In this cohort, the ROSC and early survival rates were relatively high, likely due to the large proportion of witnessed and in-hospital cardiac arrests, as well as rapid CPR initiation. A majority of survivors had favorable neurological outcomes, emphasizing the importance of prompt and effective resuscitation practices in improving survival and neurological recovery.

## Introduction

In-hospital cardiac arrest (IHCA), including events in emergency departments (EDs), is reported to occur at a rate of approximately 1.2 to 9.7 cases per 1,000 hospital admissions, highlighting the critical importance of early recognition and intervention within healthcare settings [1]. Despite advances in cardiopulmonary resuscitation (CPR), survival outcomes remain poor, with less than 10% of patients surviving to hospital discharge with a favorable neurological status [2]. In high-income countries, improved systems of care such as timely recognition, access to emergency medical services (EMS), and structured post-resuscitation interventions have contributed to modest gains in survival [3]. However, these improvements are not uniformly realized in low- and middle-income countries (LMICs), where limitations in healthcare infrastructure, emergency response systems, and trained personnel persist [4-7].

India presents unique challenges in the management of cardiac arrest. Delayed EMS response, limited availability of defibrillators, and inadequate training in advanced life support contribute to higher mortality rates [8]. Prehospital care systems remain underdeveloped across much of the country, often leading to significant delays in initiating CPR or defibrillation [9]. While global literature emphasizes the importance of factors such as initial cardiac rhythm, timing of CPR, and post-arrest care, there is a lack of comprehensive data from India assessing their impact on patient outcomes [10,11]. The absence of national cardiac arrest registries further hampers the ability to develop targeted interventions or improve clinical protocols [12,13].

This study aims to address these gaps by evaluating cardiac arrest outcomes in the ED of a tertiary care center in South India. Analyzing survival rates, neurological outcomes, and the timeliness of interventions, the study seeks to generate practical insights that can inform improvements in resuscitation practices, staff training, and resource distribution within similar healthcare environments.

The primary objective is to determine the survival rates of patients who experience cardiac arrest in the ED, including survival to hospital admission and discharge. Secondary objectives include evaluating the length of hospital stay and assessing neurological outcomes at discharge using standardized clinical measures.

## Materials and methods

Study design

This was a retrospective observational study conducted in the ED of SRM Medical College Hospital and Research Centre, Kattankulathur, Tamil Nadu, India. The study reviewed medical records of patients who experienced cardiac arrest over a 10-month period, from August 2024 to May 2025, following approval from the Institutional Ethics Committee.

Study population

The study included 240 adult patients aged ≥18 years who experienced cardiac arrest within the ED during the study period. Although some patients were later transferred to the intensive care unit (ICU), only those who arrested while in the ED were included.

Inclusion and exclusion criteria

Patients were eligible for inclusion if their medical records documented a cardiac arrest event within the ED, including the time of arrest and initiation of resuscitative maneuvers, between August 2024 and May 2025. Patients were excluded if they had pre-existing do-not-resuscitate (DNR) or do-not-intubate (DNI) orders or incomplete or missing medical records, or if they had been transferred from outside hospitals following post-arrest care.

Data collection

Data were extracted retrospectively from the hospital’s electronic medical record (EMR) system. A structured proforma was used to collect information on patient demographics such as age, sex, and comorbidities. Clinical variables included whether the arrest was witnessed, the initial rhythm (shockable or non-shockable), and the specific location of the arrest within the ED. Resuscitation details, including the time from arrest to CPR initiation, duration of resuscitation, and whether return of spontaneous circulation (ROSC) was achieved, were recorded. Outcomes included survival status, Glasgow Coma Scale (GCS) at discharge, and the need for mechanical ventilation.

Outcome measures

The primary outcome was survival beyond 48 hours following the cardiac arrest event. Secondary outcomes included neurological status at discharge, assessed using the GCS, and the total length of hospital stay among survivors.

Ethical considerations

Ethical approval was obtained from the Institutional Ethics Committee of SRM Medical College Hospital and Research Centre on August 11, 2024 (REG. No: ECR/8918/INST/TN/2013/RR-19). As the study was retrospective in nature and involved anonymized clinical data, the requirement for informed consent was waived. All data were managed in accordance with institutional policy and the ethical principles outlined in the Declaration of Helsinki.

Statistical analysis

Descriptive statistics were used to summarize patient demographics and clinical characteristics. Categorical variables were reported as frequencies and percentages, while continuous variables were expressed as means with standard deviations or medians with interquartile ranges, depending on distribution using the SPSS 26 software (IBM Corp., Armonk, NY, US). The chi-squared test was used to compare categorical variables. For continuous data, the independent t-test or Mann-Whitney U test was applied, as appropriate. A p-value of less than 0.05 was considered statistically significant.

Source of bias 

Systematic extraction from hospital records and the use of a structured data collection process minimized potential sources of selection or reporting bias. Potential sources of bias were minimized through systematic data extraction and standardized procedures; however, inherent limitations of retrospective designs cannot be fully excluded.

## Results

A total of 240 patients who experienced cardiac arrest in the ED were included in the study. The cohort demonstrated a predominance of male patients (n = 160, 66.6%) compared to female patients (n = 80, 33.4%). Most cardiac arrests were witnessed events (n = 225, 93.8%) and occurred in a hospital setting (n = 225, 93.8%), primarily within the emergency room. Following resuscitation in the ED, a subset of patients (n = 107, 44.6%) were subsequently transferred to the ICU for continued post-resuscitation care. This subset was included in the analysis because the arrest occurred prior to ICU admission, under the same continuum of emergency care initiated in the ED. It is pertinent to note that all arrests occurred in the ED prior to ICU admission, where applicable.

The initial cardiac rhythm was non-shockable in 200 patients (83.3%), predominantly asystole, while shockable rhythms such as ventricular fibrillation or pulseless ventricular tachycardia were present in 40 patients (16.7%). Cardiac causes were slightly more common than non-cardiac causes (n = 131, 54.6% vs. n = 109, 45.4%).

Return of spontaneous circulation

ROSC was achieved in 154 patients (64.1%), whereas 86 patients (35.9%) did not attain ROSC. The mean duration of CPR among the study population was 17.3 minutes, with a median of 14 minutes (interquartile range: 10-22 minutes), as documented from time-stamped electronic health records maintained during resuscitation events. These figures highlight the importance of early recognition and rapid resuscitation in improving the chances of ROSC (Table 1).

**Table 1:** Return of Spontaneous Circulation (ROSC)

ROSC achieved	n (%)
Yes	154 (64.1)
No	86 (35.9)

Time to CPR initiation

In 156 patients (65.0%), CPR was initiated within one minute of cardiac arrest, while 84 patients (35.0%) experienced a delay of two to three minutes. These findings suggest that the majority received prompt intervention, which may have positively influenced outcomes (Table 2).

**Table 2:** Time From Cardiac Arrest to CPR Initiation Significant p-value < 0.05 CPR: cardiopulmonary resuscitation

Time interval	n (%)	p-value	Chi-squared value
0–1 minute	156 (65.0)	0.0013	10.35
2–3 minutes	84 (35.0)		

Survival beyond 48 hours

Survival beyond 48 hours post-arrest was observed in 154 patients (64.1%), while 86 patients (35.9%) did not survive beyond this time point. This suggests relatively favorable short-term survival following resuscitation efforts in the study population (Table 3).

**Table 3:** Survival Status at 48 Hours

Survival status	n (%)
Survived	154 (64.1)
Did not survive	86 (35.9)

Length of hospital stay

Length of hospital stay was recorded in only 81 patients (33.7%). Among them, 67 patients (27.9%) had a stay ranging from one to nine days, and 13 patients (5.4%) had a documented stay of 17 days. One case (0.4%) reported a stay from January 22 to February 12, ending in death. The majority of cases (n = 159, 66.3%) had no available data on hospital stay, indicating documentation gaps (Table 4).

**Table 4:** Length of Hospital Stay Significant p-value < 0.05

Duration (days)	n (%)	p-values	Chi-squared values
1-9	67 (27.9)	0.000001	115.68
9-17	13 (5.4)		
>17	1 (0.4)		
Not recorded	159 (66.3)		

Neurological status and disability at discharge

GCS scores at discharge were documented for all patients. A normal GCS of 15/15 was recorded in 178 patients (72.1%), suggesting intact neurological function in a majority of survivors. Lower scores were also noted: GCS 10/15 in 12 patients (4.9%), and severely depressed scores such as 2T/15 and E1 V1 M1 in 13 patients each (5.3%). In terms of disability at discharge, 26 patients (10.8%) required mechanical ventilation, indicating persistent neurological or respiratory compromise, while 214 patients (89.2%) were discharged without documented disability (Table 5).

**Table 5:** GCS and Disability at Discharge Significant p-value < 0.05 GCS: Glasgow Coma Scale

GCS at discharge	n (%)	p-values	Chi-squared values
15/15	178 (72.1)	0.000001	379.67
2T/15	13 (5.3)		
E1 V1 M1	13 (5.3)		
10/15	12 (4.9)		

Disability at discharge	n (%)	p-values	Chi-squared values
On mechanical ventilation	26 (10.8)	0.000001	147.27
No disability	214 (89.2)		

## Discussion

Summary and interpretation of key findings

This retrospective study investigated clinical outcomes following cardiac arrest and resuscitation in the ED of a tertiary care center in South India. Among 240 patients analyzed, the majority were male (n = 160, 66.6%), and most arrests were witnessed (n = 225, 93.8%). Non-shockable rhythms predominantly asystole were observed in 83.3% (n = 200), with only 16.7% (n = 40) presenting with a shockable rhythm. ROSC was achieved in 154 patients (64.1%), and an equal proportion survived beyond 48 hours. These findings indicate comparatively favorable short-term outcomes, as survival beyond 48 hours in our study (64.1%) exceeded the commonly reported rates of 30%-50% in similar LMIC settings [1-3]. These outcomes also reflect growing efforts in LMICs to develop prehospital and EMS, as seen in recent African and South Asian analyses that underscore the need for scalable systems and emergency access readiness [14,15].

Sex-based disparities and clinical significance of witnessed arrests

The predominance of male patients aligns with findings from Peberdy et al. and Benjamin et al., which attribute this disparity to higher baseline cardiovascular risk among men [2,6]. In addition, the high rate of witnessed cardiac arrests (93.8%) is clinically significant. Literature indicates that witnessed arrests allow for faster initiation of CPR, thereby improving survival and neurological outcomes [4,7]. These results reflect the benefit of trained clinical staff and suggest potential value in expanding basic life support (BLS) training to bystanders in public settings.

Impact of resuscitation timing on ROSC and early survival

Timely initiation of CPR within one minute of cardiac arrest was documented in 156 patients (65.0%), likely contributing to the relatively high ROSC rate (64.1%). Meaney et al. and Jentzer et al. have consistently shown that early, high-quality CPR correlates strongly with favorable short-term survival, particularly in cases where intervention occurs within three minutes of arrest onset [5,10]. Nonetheless, 86 patients (35.9%) did not achieve ROSC, underscoring the clinical limitations in managing non-shockable rhythms; a finding echoed by Andersen et al. in their study of IHCAs [16].

Short-term survival outcomes in an LMIC setting

The 48-hour survival rate of 64.1% observed in this study exceeds many regional and international reports from LMICs. For example, Irfan et al. documented considerably lower survival in a national out-of-hospital cardiac arrest (OHCA) registry from Qatar, attributing this to systemic delays and variable prehospital care [17]. Similarly, studies from South Asia and sub-Saharan Africa report early survival rates under 50%, particularly in resource-constrained environments [18,19]. While encouraging, our findings are limited to early survival; survival to discharge and long-term functional recovery were not fully assessed.

Neurological recovery and post-resuscitation support

Neurological status at discharge was favorable in most patients. Among survivors assessed at discharge, a GCS score of 15/15 was recorded in 178 patients (72.1%), while 26 patients (10.8%) required mechanical ventilation. These results align with literature emphasizing the importance of early ROSC and aggressive post-arrest care in mitigating hypoxic brain injury [11,20]. However, 66.3% (n = 159) of cases lacked complete length-of-stay data, suggesting deficiencies in record-keeping and limiting accurate assessment of rehabilitation needs. This is consistent with observations by Adhikari et al. regarding gaps in clinical documentation across Indian critical care units [13].

Comparative insights with global data

Globally, survival outcomes and arrest rhythms vary significantly by region. Girotra et al. reported a significant increase in survival of IHCA between 2000 and 2009 in the United States of America, whereas Wissenberg et al. demonstrated improvements tied to coordinated national protocols in Denmark [1,3]. Our findings, including the high prevalence of non-shockable rhythms and moderate ROSC rates, are comparable to data from low-resource settings, where defibrillator access and Advanced Cardiovascular Life Support (ACLS)-trained personnel are often limited [18,21]. Notably, our results illustrate that, with rapid response systems in place, even resource-limited facilities can achieve early outcomes comparable to those in higher-income countries [22,23]. Compared to high-income countries with established prehospital systems, survival outcomes in this study align more closely with other LMICs due to similar systemic limitations and response frameworks.

Study limitations

This study had several limitations. First, its retrospective design is susceptible to selection and information bias due to incomplete documentation. Second, the lack of follow-up beyond 48 hours limits our understanding of long-term neurological function and survival to discharge. Third, the absence of a centralized national cardiac arrest registry in India restricts the generalizability of the data. These constraints are consistent with observations from Checkley et al. and Adhikari et al., who call for standardized protocols and data infrastructure in LMICs [13,24].

Future directions and systemic recommendations

To improve outcomes, institutional and national efforts should prioritize strengthening documentation, developing cardiac arrest registries, and promoting public BLS and ACLS training. Targeted post-arrest strategies such as temperature management and early coronary angiography also warrant exploration in future trials, particularly given their cost-effectiveness in LMIC settings [25,26]. Furthermore, integration of low-cost, evidence-based interventions should be a strategic focus to reduce disparities in cardiac arrest outcomes.

## Conclusions

This study provides important insights into cardiac arrest outcomes within the ED of a tertiary care center in South India. Among 240 patients analyzed, ROSC was achieved in 64.1% (n = 154), with the same proportion surviving beyond 48 hours, suggesting moderately favorable short-term outcomes. However, the predominance of non-shockable rhythms (83.3%, n = 200) and variability in neurological recovery at discharge reflect ongoing challenges in cardiac arrest management. The high rate of witnessed arrests (93.8%, n = 225) indicates a critical window for improving survival through prompt intervention, highlighting the need for widespread public training in BLS.

Despite these findings, variability in neurological outcomes and incomplete follow-up data point to the need for standardized documentation and structured post-arrest care. Future research should focus on long-term survival, neurological function, and quality of life post-arrest, especially in resource-limited settings. The potential benefits of integrating advanced resuscitation strategies such as targeted temperature management and early coronary interventions also warrant exploration. Establishing national cardiac arrest registries in India is essential, not optional for benchmarking outcomes, developing evidence-based clinical protocols, and driving informed policy decisions to improve survival.
